# Homesick: residential and care patterns in patients with severe mental illness

**DOI:** 10.1186/s12888-016-1137-6

**Published:** 2016-12-03

**Authors:** Liselotte D. de Mooij, Martijn Kikkert, Nick M. Lommerse, Jan Theunissen, Mariken B. de Koning, Lieuwe de Haan, Aartjan T. F. Beekman, Pim W. R. A. Duurkoop, Jack J. M. Dekker

**Affiliations:** 1 Arkin Research Department, Klaprozenweg 111, 1033 NN Amsterdam, The Netherlands; 2 GGZ inGeest Research Department, Arent Janszoon Ernststraat 1187, 1081 HL Amsterdam, The Netherlands; 3 Arkin Department Mentrum, Baron G.A. Tindalstraat 27, 1019 TS Amsterdam, The Netherlands; 4 Academic Medical Centre Department Early Psychosis, Meibergdreef 5, 1105 AZ Amsterdam-Zuidoost, The Netherlands; 5 Vrije Universiteit Medical Centre Department Psychiatry, De Boelelaan 1117, 1081 HV Amsterdam, The Netherlands; 6 GGZ inGeest, Arent Janszoon Ernststraat 1187, 1081 HL Amsterdam, The Netherlands; 7 Vrije Universiteit Department Clinical Psychology, Van der Boechorststraat 1, 1081 BT Amsterdam, The Netherlands

**Keywords:** Changes of residential setting, Changes of care setting, Changes in severe mental illness, Address changes, Hospitalisation, Revolving door

## Abstract

**Background:**

Changes in the residential and care settings of patients with severe mental illness (SMI) are a concern because of the large variety of possible negative consequences. This study describes patterns of changes in the residential and care settings of SMI patients and explores associations between these changes, sociodemographics, and clinical characteristics.

**Methods:**

From January 2006 to January 2012, all data relating to changes in residential and/or care setting by SMI patients (*N =* 262) were collected from electronic case files. Data covering psychopathology, substance use, and medication adherence were assessed in 2006.

**Results:**

There were more changes in the residential than in the care setting. In 6 years, only 22% of our sample did not move, 23% changed residence once, 19% twice, 10% three times, and 26% four or more times. Substance use predicted changes of care and/or residential setting and rehospitalisation. The severity of negative symptoms predicted rehospitalisation and duration of hospitalisation. Disorganisation symptoms predicted the duration of hospitalisation.

**Conclusions:**

A majority of patients with SMI changed residential and/or care settings several times in 6 years. Patients with substance use or severe negative and disorganisation symptoms may need more intensive and customised treatment. Further research is needed to investigate prevention programmes for highly-frequent movers.

## Background

The mental health-care system aims to allocate patients to the most appropriate house setting where they can stay for a long time. Although this seems straightforward, studies show that patients change address frequently [1–3]. Residential stability, the frequency in which one changes address, is an important determinant of quality of life in people with SMI and it is often a pre-condition for effective treatment and rehabilitation [4–6]. Instability in this domain is therefore stressful, and it can disrupt the continuity of treatment and cause social isolation [4, 7] or relapse [8].

Residential changes are often a consequence of a change in care setting. Care setting refers to the type of care and can be generally divided into outpatient care for independently living patients, sheltered housing, and psychiatric admission. Changing from one care setting to another usually involves changing residence too. Allocating patients to another care setting is related to changing personal needs and abilities, but may also be associated with efforts to cut psychiatric beds in mental health care, which is an important goal in the Western world. Sheltered housing and outpatient care are used as alternatives to long-term psychiatric admission [9–12]. This is demonstrated by the fact that the Netherlands, with a population of 160,000 SMI patients, has 11,427 places in sheltered housing facilities and 18,499 in clinical care facilities [13, 14]. The Netherlands therefore has much more intramural capacity than other Western countries [14].

Most SMI patients in Western countries live independently, either sharing a house with others such as relatives or a partner or living alone [15]. In general, patients who live independently receive outpatient treatment in an integrated approach comprising psychological and psychiatric treatment, and supported employment where possible. The primary treatment goals are the stabilisation of symptoms, preventing acute relapse and psychiatric hospitalisation, and improvements in social inclusion and structural activities. If independent housing is not feasible, SMI patients rely on residential care in sheltered housing. Sheltered housing often provides a range of housing options and care intensity [16]. If possible, patients are guided into independent housing; otherwise, the goal is to stabilise patients in long-term sheltered housing [17]. When sheltered housing is not an option, admission to a psychiatric hospital will be necessary. If these patients recover, independent housing or sheltered housing is encouraged [18].

Some studies have examined the risk factors for highly-frequent hospital admissions and discharges: “revolving door” cases [19]. The leading risk factors are being male [20], being younger [1, 21], substance abuse, and medication non-adherence [19, 22]. However, there is a relative paucity of studies looking at the link between health and changes in the setting because of a lack of detailed information about health status and residential changes over time [23], particularly for patients with SMI [2]. Many studies have limited follow-up periods of only 2 years or less [5]. Furthermore, these studies often report the number of changes but failed to provide information about stay duration or longer-term patterns [1]. It is important to understand when and why the mental health-care system succeeds in providing patients with a stable home situation. A clearer picture will allow us to adapt our treatment and prevent frequent moves by SMI patients.

This study describes patterns of changes in care settings and in residential mobility in SMI patients over a follow-up period of 6 years. Secondly, we explored potential sociodemographics and clinical risk factors of (a) changes in care setting and (b) residential changes, (c) number and (d) total duration of psychiatric admissions. Hypotheses on patterns of change were based on policy and objectives of the different care settings, and on opinions of experts in this field such as clinicians and policy makers. This allowed us to specify the following patterns in care setting movements during the 6 years follow up period: (a) 25% of patients in sheltered housing will move to independent housing, (b) 65% of patients in sheltered housing will be long-term residents, and (c) 80% of inpatients will move to sheltered housing or to independent housing. Finally, we expected patients with more severe psychopathology and higher indices of substance use to be more likely to change residence and/or care setting more often. To the best of our knowledge, this is the first study to examine changes of residence, the relationship with the care setting and the associated determinants.

## Methods

This longitudinal study conducted between 2005 and 2011 was based on a survey sample of SMI patients treated by the mental health-care institutions Arkin, GGZ InGeest, RIBW and HVO Querido in Amsterdam (Netherlands). These institutions are jointly responsible for the treatment of SMI patients residing in Amsterdam. The principal objectives of the study were to evaluate changes in quality of life, disease characteristics, general functioning, care needs, social networks and inclusion in society, and victimisation [24]. The current study is based on data relating to patient movements between 01/01/2006 and 01/01/2012. Data for severity of psychopathology, quality of life, substance use, and medication adherence were assessed at baseline in 2006. The study was approved by the Dutch Association of Medical-Ethical Appraisal Committees (NVMETC) for mental-health organisations.

### Population, inclusion and exclusion criteria

Included patients fulfilled the criteria for a DSM-IV diagnosis of schizophrenia, psychotic disorder, substance use, severe mood or anxiety disorders and a history of intensive mental health care during the previous 2 years. Further inclusion criteria were: adequate mastery of Dutch or English and residence in the Amsterdam district for at least one year. Exclusion criteria included being unable to understand questions or communicate, or being unable or unwilling to give informed consent. Patients with comorbid substance use disorders were also included when they fulfilled these criteria [25]. The attending psychiatrist made the diagnoses.

### Enrolment

In this study, 876 patients were randomly selected from 2846 patients treated by outpatient teams, or in sheltered housing facilities and inpatient care facilities. The aim was to include equal numbers of patients from the three care settings. A total of 553 patients (63.1%) were not included in the study. Some patients refused to participate (25.9%); others did not participate for unknown reasons (25.5%). Another 2.9% of the patients were excluded because their clinicians deemed that inclusion would affect their clinical status. Patients who were no longer receiving treatment (8.9%) were also excluded. The remaining 323 patients (37% of the randomly-selected sample) were included in the study [24]. Once these patients had given written informed consent, the assessment was performed in a face-to-face interview. The interviews were conducted at the centre for mental health care or, if preferred by the patient, at the patient’s home. The interview took one and a half hours and the patients received €15. The interviews were conducted by a trained psychologist, research assistants, and a senior researcher. The patients included were assessed again for the purposes of follow-up 6 years after inclusion.

### Measures

Data about residential mobility and care setting came from electronic case files. Case notes were examined by a psychologist and a senior researcher to identify all address changes between 01/01/2006 and 01/01/2012, and all changes in care settings; living independently, sheltered housing and psychiatric hospital. Changes in the residential setting were defined as physical moves to another address, or a transfer to another department or ward within a housing facility such as a sheltered housing accommodation or a psychiatric hospital. Temporary moves, lasting less than 14 days, were not recorded as a change in address or care setting.

Patients can change address but remain in the same care setting. For instance, a patient may move from one sheltered housing facility to another. Moves of this kind were registered as a residence change but not as a change in care setting.

Severity of psychopathology was measured with the Brief Psychiatric Rating Scale-Expanded (‘BPRS-E’) [26, 27], which consists of 24 symptoms assessed on a scale from 1 to 7. Items are grouped in four subscales - positive symptoms, negative symptoms, depression and disorganisation [27] - and scored on the basis of observations during the interview and patient self-reports. The BPRS-E is a sensitive instrument with good inter-rater reliability (*r =* 0.74, *p* < .001) and validity [26, 28, 29].

Use of alcohol and drugs, substance dependence and abuse were assessed with the Measurements in the Addictions for Triage and Evaluation, or MATE [30], which assesses the use of psychoactive substances, lifetime and current substance abuse and dependence on the basis of DSM-IV [31]. The MATE is a valid instrument: inter-rater reliability ranges between 0.75 and 0.92, and interviewer reliability ranges from 0.34 to 0.73 [30]. Patient files were also consulted to identify patients with a DSM-IV diagnosis of substance abuse or dependence. Patients were classified as having a dual diagnosis when the MATE indicated substance abuse or dependence or when a patient had already been diagnosed with a substance use disorder.

Medication adherence was assessed with the Medication Adherence Questionnaire (MAQ). The MAQ consists of four yes/no questions about ways in which patients may fail to take their prescribed medication: forgetting, carelessness, stopping the medication when they feel better and or stopping the medication because they believe it makes them feel worse [32]. Patients with a score ≤ 3 on the MAQ were defined as non-adherent [32–34]. The MAQ is a valid and reasonable instrument for detecting non-adherence [32, 35].

### Data analysis

Analyses were conducted using SPSS 22 (SPSS Inc., 2009). Frequency distributions were used to describe the data, chi-square analyses were used for categorical variables and ANOVA analyses were used to analyse continuous variables. When the expected cell count was too low to perform a chi-square test, Fisher’s exact test was used. A Kruskal-Wallis test was used when ANOVA assumptions were violated and a Mann-Whitney test was used when *t*-test assumptions were violated. Generalised linear regression models (BACKSTEP method) were used to derive prediction models for the following three dependent variables: number of admissions, residential movements, and number of hospitalisation days. This analysis was considered appropriate since our data do not match the assumptions for multivariate linear regression models. The independent sociodemographic and clinical variables were age, gender, ethnicity, education, diagnosis, dual diagnosis, medication adherence, positive symptoms, negative symptoms, depression and anxiety, disorganisation, alcohol, cannabis, and hard drugs. Independent variables were removed stepwise during the statistical analyses when they did not contribute to the fit.

## Results

The SMI cohort included 323 patients in 2006. In 2012, patient files were not available or incomplete for 49 patients. Data for six patients were not included because these patients were homeless for a longer period of time and another six patients were excluded because they did not give permission for the use of their patient files. As a result, complete data about residential and care setting changes were available for 262 patients. Patients were grouped into the categories ‘living independently’, ‘sheltered housing’ and ‘psychiatric hospital’ on the basis of their situation on 01–01–2006. In this sample, 100 patients were in a psychiatric hospital at baseline (87% in a long-stay facility, 11% in a short-stay facility and 2% in an acute facility). Ninety per cent of the patients living in sheltered housing lived there with ten or more patients and only 10% lived with nine patients or fewer. Table 1 provides a summary of the main sociodemographic and clinical characteristics.

**Table 1:** Socio-demographics, care facilities and clinical characteristics of the study sample (*N* = 262)

	Independent housing (*n* = 103)		Sheltered housing (*n* = 59)		Psychiatric hospital (*n* = 100)		*χ* ^2^/*P* ^*a*^
Socio-demographics							
Age, mean (*SD*)	46.1	(10)	45.7	(10.5)	43.8	(11)	0.261
Gender, *n* (*%*)							
Male	53	(51.5)	40	(67.8)	69	(69)	*0.021*
Female	50	(48.5)	19	(32.2)	31	(30)	
Ethnicity, *n* (*%*)							
Western	72	(69.9)	30	(50.8)	61	(61.6)	0.055
Non-Western	31	(30.1)	29	(49.2)	38	(38.4)	
Education, *n (%)* ^b^							
Primary/secondary education	72	(69.9)	50	(84.7)	82	(82)	
Higher education	25	(24.3)	8	(13.6)	8	(8)	*0.006*
Unknown	6	(5.8)	1	(1.7)	10	(10)	
Diagnosis, *n* (*%*)^b^							
Schizophrenia and other psychotic disorders	72	(69.9)	49	(83.1)	95	(95)	
Mood disorders/anxiety disorders/axis II disorders	21	(20.4)	3	(11.5)	2	(7.7)	*0.000*
Substance use disorders	10	(9.7)	7	(11.7)	3	(3)	
Comorbid substance use disorders, *n* (*%*)	21	(20.4)	27	(45.8)	22	(22)	*≤0.001*
Medication non-adherence	38	(40)	28	(51.9)	40	(44)	0.375
Symptoms (BPRS-E), mean (*SD*)^c^							
Positive symptoms	1.6	(0.8)	1.5	(0.6)	2.1	(1.0)	*0.000*
Negative symptoms	1.2	(0.3)	1.3	(0.4)	1.7	(0.7)	*0.000*
Depression & Anxiety	1.8	(0.8)	1.7	(0.6)	2.0	(0.8)	0.067
Disorganisation	1.2	(0.2)	1.3	(0.4)	1.6	(0.6)	*0.000*
BPRS total	1.4	(0.4)	1.4	(0.4)	1.8	(0.5)	*0.000*
Substance use (MATE), *mean* (*SD*)^c,d^							
Alcohol	5.4	(10.2)	8.5	(12.4)	4.7	(10)	0.362
Cannabis	2.3	(7.2)	6.2	(11.3)	3.4	(8.9)	*0.007*
Hard drugs (cocaine, stimulants, 3,4-methylenedioxy- methamphetamine, opiates)	1.7	(7)	5.1	(12.1)	1.3	(7.1)	*0.001*

BPRS-E, Brief Psychiatric Rating Scale – Expanded; *MATE* Measurements in the Addictions for Triage and Evaluation

^a^P is the result of ANOVA for continuous variables and *χ* ^2^ test for categorical variables. Significant findings at *P* = 0.05 or less are shown in italics

^b^Expected cell count too low (<5) for accurate chi-square, Fisher’s exact test was performed

^c^Assumptions of ANOVA were violated, Kruskal-Wallis test was performed. Number of days of substance use in the last month

As expected, the different care settings differed (*P* < .05) at baseline in terms of diagnosis, symptom severity, gender, education, diagnosis, cannabis and hard-drug use. However, standardised residual values showed no significant discrepancies for the different groups or for gender. Patients living independently were more highly educated than patients in a psychiatric hospital or sheltered housing (*χ* ^2^(4) = 13.9), and these patients were also more likely to have mood disorders, anxiety disorders or axis II disorders than inpatients (*χ* ^2^(4) = 7.5). Patients in sheltered housing were more often diagnosed with a dual diagnosis than patients living independently and patients in a psychiatric hospital (*χ* ^2^(2) = 14.2). Inpatients had more severe positive (H(2) = 16.1), negative (H(2) = 49.9) and disorganisation (H(2) = 41.9) symptoms and a higher total psychopathology score (H(2) = 31) than patients living in sheltered housing or independently. Patients in sheltered housing had more days of cannabis (H(2) = 9.8) and hard-drugs (H(2) = 15.3) use in the past month than patients living independently and patients in a psychiatric hospital.

### Changes in care setting

**Fig. 1:**
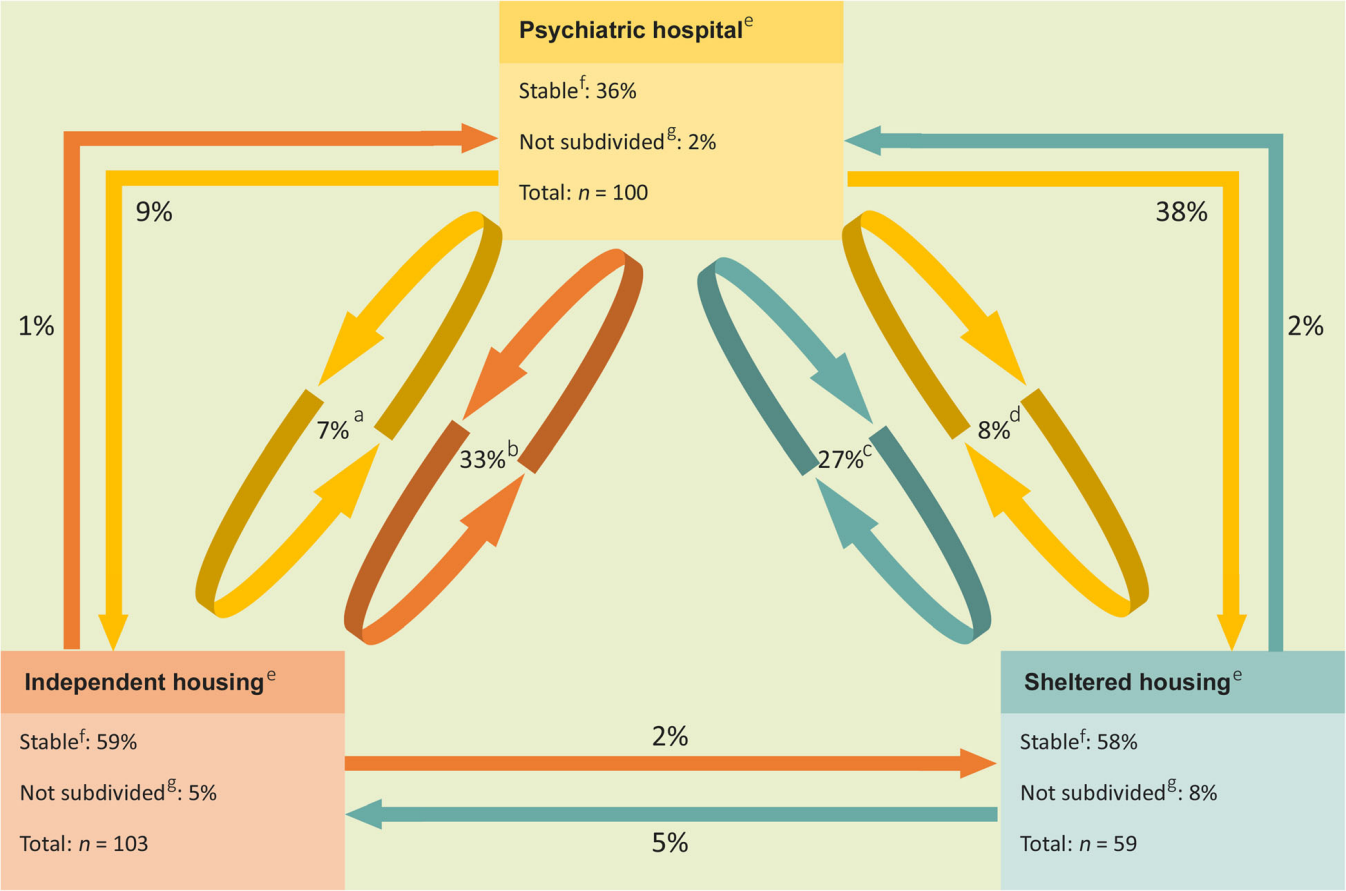
Changes between independent housing, psychiatric hospital and sheltered housing over 6 years (*N* = 262). **a** Patients moving back and forth between a psychiatric hospital and independent housing. **b** Patients moving back and forth between independent housing and a psychiatric hospital. **c** Patients moving back and forth between sheltered housing and a psychiatric hospital. **d** Patients moving back and forth between a psychiatric hospital and sheltered housing. **e** Each type of care setting counts as 100%. **f** ‘Stable’ refers to patients who lived in one type of care setting during the entire follow-up period. **g** ‘Not subdivided’ refers to patients who do not fit in any of the patterns shown in the figure

Patterns in changes of care setting during the six-year follow-up period were analysed to establish a clearer picture of how patients switch between different settings (Fig. 1). Patients were assigned to independent housing, sheltered housing, or psychiatric hospital groups on the basis of their situation on 01–01–2006. These three different settings were adopted as the starting point for every change. Residential changes within the same type of care setting were not included in these analyses. Some patients were admitted to general hospitals for somatic disorders (*n =* 30). These movements were not classified as changes in care setting.

We studied the movement patterns between three different types of care setting in each of the three groups described above. Patients who lived in one type of care setting throughout the follow-up period were considered stable. As clearly shown in Fig. 1, the patterns are similar in some of the groups described above: the group of patients switching between independent housing and a psychiatric hospital and patients switching between a psychiatric hospital and sheltered housing. Consequently, we conclude that there are six main patterns of changes in care setting: 1. living independently throughout the follow-up period, 2. sheltered housing, 3. a psychiatric hospital, 4. switching between a psychiatric hospital and living independently, 5. switching between psychiatric hospitals and sheltered housing and 6. moving from psychiatric hospitals to sheltered housing. Over a period of 6 years, half the patients stayed in the same care setting. Twenty-one per cent of SMI patients changed care setting once, 11% twice, 5% three times, and 14% four or more times.

Residential changes in were more frequent than changes in care setting: 78% changed address one or more times. Twenty-three per cent of patients with SMI changed address once, 19% twice, 10% three times and 26% four times or more. For each of the the six main patterns derived from Fig. 1, we looked at changesresidence, hospitalisation days, and number and type of admissions (Table 2). Patients moving back and forth between different care settings were included as a single group. These patients had the highest rates for residence changes, number of admissions and acute admissions.

**Table 2:** Changes in residence, hospitalisation days, number and type of admissions over 6 years (*N* = 262)^a^

		Residential changes^b^			Number of admissions			Hospitalisation days			Type of admission^c^		
											Acute stay	Short stay	Long stay
	*N*	Mean	Range	*SD*	Mean	Range	*SD*	Mean	Range	*SD*	*%*	*%*	*%*
Patients in independent housing (stable pattern)^d^	61	0.7	0–8	1.4	0	0	0.0	0	0	0.0	0	0	0
Patients in a psychiatric hospital (stable pattern)^d^	36	2.9	0–8	2.0	1.1	1–2	0.3	1934	0	499.7	8.3	30.6	100
Patients in sheltered housing (stable pattern)^d^	34	0.9	0–2	0.8	0	0	0.0	0	0	0.0	0	0	0
Patients moving back and forth between different care settings	65	5.6	1–20	4.1	2.7	1–8	2.0	504.7	1–2076	646.2	60	72.3	32.3
Patients moving from psychiatric hospitals to sheltered housing	38	1.7	1–4	1.0	1	1–2	0.2	1365.1	318–2101	516.0	5.3	2.6	100
Patients moving from psychiatric hospitals to independent housing	9	1.8	1–2	0.4	1.1	1–2	0.3	1298.2	437–1977	531.8	57.1	100	100
All patients	262	2.8	0–20	3.4	1.1	0–8	1.5	662.2	0–2191	826.3	18.7	27.5	42.4

^a^Changes in care setting affecting fewer than 6% of patients are not presented in this table

^b^Moves to other address, changes in the type of care setting, or moves to another department in the same care setting were considered to be changes in the residential setting

^c^The percentages are the proportion of patients with acute admissions, or patients admitted for short or long stays in psychiatric hospitals

^d^‘Stable’ refers to patients who spent the entire follow-up period in one type of care setting

To improve our understanding of which patients are more likely to change care setting and residence frequently, we performed four generalised linear model (GLM) analyses (Table 3) to predict changes in care setting, residential changes, number of admissions, and days in a psychiatric hospital. More frequent changes in the type of care setting were found in younger patients with a Western background and substance use in the last month. Patients with more admissions were younger and had a Western background, substance use disorders, and fewer negative symptoms. Finally, younger patients with fewer mood disorders/anxiety disorders or axis II disorders, with less medication non-adherence, with more negative and disorganisation symptoms, and with less alcohol and hard drugs use in the last month were more likely to stay longer in psychiatric care facilities.

**Table 3:** Results of GLM analyses for predictors of changes in care setting, residential changes, number of admissions, and days in a psychiatric hospital between 01/01/2006 and 01/01/2012 (*N* = 262)

					95% CI for Exp(B)^c^	
	B	*S.E.*	*P*	Exp B	Lower	Upper
Dependent factor: Changes in care setting^a,1^						
Included						
Age	-0.025	0.0082	*0.002*	0.975	0.960	0.991
Ethnicity						
Non-Western	-0.536	0.1784	*0.003*	0.585	0.412	0.830
Western	ref					
Substance use disorders	1.159	0.2722	*0.000*	3.188	1.869	5.435
Schizophrenia and other psychotic disorders	ref					
*Intercept*	1.603	0.3852	*0.000*	4.969	2.335	10.572
Dependent factor: Residential changes^b,2^						
Included						
Substance use disorders	0.653	0.2582	*0.011*	1.921	1.158	3.186
Schizophrenia and other psychotic disorders	ref					
*Intercept*	0.936	0.0803	*0.000*	2.551	2.180	2.986
Dependent factor: Number of admissions^3^						
Included						
Age	-0.023	0.0106	*0.028*	0.977	0.957	0.998
Ethnicity						
Non-Western	-0.566	0.2329	*0.015*	0.568	0.360	0.896
Western	ref					
Substance use disorders	0.977	0.3301	*0.003*	2.657	1.391	5.075
Schizophrenia and other psychotic disorders	ref					
Negative symptoms (BPRS)	0.453	0.1914	*0.018*	1.574	1.081	2.290
*Intercept*	-0.166	0.1492	0.265	0.847	0.632	1.135
Dependent factor: Days in psychiatric hospital^4^						
Included						
Age	-0.014	0.0067	*0.030*	0.986	0.973	0.999
Mood disorders/anxiety disorders/axis II disorders	-0.943	0.2429	*0.000*	0.389	0.242	0.627
Schizophrenia and other psychotic disorders	ref					
Medication non-adherence	-0.390	0.1576	*0.013*	0.677	0.497	0.922
Medication adherence	ref					
Negative symptoms (BPRS)	0.733	0.2077	*0.000*	2.081	1.385	3.127
Disorganisation	0.581	0.1976	*0.003*	1.788	1.214	2.634
Alcohol	-0.003	0.0014	*0.018*	0.977	0.994	0.999
Hard drugs	-0.019	0.0041	*0.000*	0.981	0.973	0.989
*Intercept*	5.569	0.4482	*0.000*	262.041	108.854	630.805

^a^Changes of care setting include all moves between independent and sheltered housing, and psychiatric hospitals

^b^Moves to other address, or moves to another department or ward in the same care setting were considered to be changes in the residential setting

^c^CI = confidence interval

^d^Significant findings at *P* = 0.05 or less are shown in italics

^1^Omnibus test; *P* = 0.000, ^2^Omnibus test; *P* = 0.024, ^3^Omnibus test; *P* = 0.001, ^4^Omnibus test; *P* = 0.000

## Discussion

This study reports on unique data about patterns of changes in residential and care settings in SMI patients. Over a period of 6 years, 33% of patients living independently switched from their own homes to a psychiatric hospital, and vice-versa. Twenty-seven per cent of patients in sheltered housing were admitted at least once to a psychiatric hospital, often for a short admission. Furthermore, 15% of the inpatients switched between psychiatric hospitals, independent and sheltered housing.

We hypothesized that 25% of patients in sheltered housing would move to independent housing during the 6 years follow-up. As can be seen in Fig. 1, only 5% of patients made this move. The majority (65%) of sheltered housed patients were expected to stay in sheltered homes. Although we found a slightly lower proportion of 58%, our results confirm this hypothesis. We also hypothesized that 80% of patients in psychiatric hospitals would move to sheltered or independent housing. We found that only 47% of inpatients moved successfully to sheltered (38%) or independent (9%) housing.

The data yielded another important findings. We found patients who lived stably in independent housing (59%), sheltered housing (58%), or in psychiatric hospitals (36%). However, over the period of 6 years we studied, half of the patients failed to achieve care stability, changing care setting one or more times. Residential changes were even more frequent: almost 80% moved one or more times and these patients had an average of almost six address changes in 6 years. This is in sharp contrast with the general population, which changes address an average of once every 10 years [36]. Those patients who moved back and forth between different care settings were most likely to change residence and to have the highest number of short admissions. Bearing in mind that an admission or an address change may disrupt the continuity of care, the unfortunate conclusion is that the patients most affected are precisely those for whom continuity of care is most important. However, many patients also have favourable, stable, housing patterns.

Our results indicate that only limited numbers of patients (5%) move from sheltered to independent housing, contrary to the aims of the deinstitutionalisation process [17]. This is a remarkable finding since the goal of sheltered housing has been to encourage patients to live as independently as possible. Given our data, it can be concluded that this goal is seldom achieved. One explanatory factor could be the shortage of affordable independent housing in Amsterdam. Another factor meriting consideration is the desirability of maximising the numbers of patients moving into independent housing: it is conceivable that forcing a patient to live independently without intensifying outpatient care could exacerbate a patient’s condition.

After psychiatric admission, the majority of inpatients were expected to move (back) to independent housing or sheltered housing. There was indeed a relative large outflow (38%) of patients from a psychiatric hospital to sheltered housing. Most of these patients came from long stay departments. Moving from a psychiatric hospital to independent housing seems more difficult and was observed a lot less than expected. Only 9% of inpatients moved successfully to independent housing. Compared to inpatients who moved to sheltered housing, these patients came from an acute or short stay ward more often.

We found a relatively large group of patients living stably in psychiatric hospitals. This conflicts with the aims of deinstitutionalisation and is due to the fact that Amsterdam, although less than in other parts of the Netherlands, still has a relatively large number of psychiatric beds [14, 37]. Instigated partly by changes in policy objectives, regulations and funding, there is now consensus among policy makers that mental health care should shift more towards placing the care of psychiatric patients in the community rather than in institutions. Therefore a national innovation programme has recently been developed to improve care of severe mental illness, recovery of health, participation and personal identity, and to help people with serious mental health issues catch up with the rest of society [38].

Furthermore, our data identified modifiable predictors for frequent care and residential changes. Substance use at baseline predicted both re-hospitalisation, changes in care setting and residential changes. In addition, more severe negative symptoms predicted re-hospitalisation and longer hospitalisation. Longer hospitalisation was predicted by more severe disorganisation. These results are consistent with other studies [3, 39–41]. Medication adherence and less substance use were predictors for longer hospitalisation. We think this is a spurious finding, since psychiatric hospitals provide more supervision and guidance in the areas of medication and the restriction of substance use. Our findings also showed that patients who failed to achieve stability were more frequently admitted to short-stay hospitals than to long-stay hospitals. Revolving-door patients may be admitted for shorter periods of time when substance use or medication non-adherence are the main cause of admission. When relief from acute intoxication or the regulation of medication are achieved, most patients will be ready to leave the hospital after a short period [19].

### Limitations

This study had several limitations. First, the observational design means that we cannot demonstrate causation. The other issue is that we included only patients who receive psychiatric care; this may have resulted in the underestimation of the prevalence of changes. Moreover, we excluded patients who were homeless because we could not track them during the six-year study period. Homeless patients change care setting most frequently. More than half of the randomly selected patients were not included in our study. High dropouts are a common problem in studies with psychiatric patients and can reduce the external validity. We believe that those who were not included will generally be the more severely ill patients and they may change care and residential setting even more often. Furthermore, inpatients were over-represented in our sample group (38%) by comparison with the general SMI population (13%) [15]. Our results may therefore not be representative for the general SMI population. Despite our elaborate assessment of all changes in care and residential setting as registered in patient files, some patient files may have been incomplete. Incomplete information about addresses and accommodation status codes are a feature of routine clinical records [3].

## Conclusions and implications

Our study shows that half the patients underwent multiple changes of care setting in 6 years. Approximately half the patients in each subgroup were stable in terms of the type of care setting. However, this does not mean that they always lived at the same address. Patients living stably in psychiatric hospitals and patients moving back and forth between different care settings changed residence most frequently.

Residential instability may be the consequence of how mental health care is usually organized. For each care setting different housing facilitites have been realized in most western countries. Consequently, if a patient’s needs change and a different form of housing is required, one needs to move to another housing facility on another address. Approximately half of patients with schizophrenia will have an episodic course [42] which may result in many address changes over time. A care system which is able to provide a wider range of psychiatric and housing support on the same address, would make it possible for patients to stay in one place longer. There are some developments in this direction such as Intensive Home Treatment which allows patients to stay in their homes during crisis instead of being admitted to hospital [43].

Unstable residential and care patterns were predicted by substance use and the severity of negative symptoms. It is important to note that frequent moves are an underestimated stress factor and contribute to psychological instability. Adapting to new living conditions may contribute to stress, estrangement and an impoverishment of the social network [39]. Our secondary analysis did indeed confirm that patients with more re-hospitalisations had less frequent contact with people in their social network.

Given the ongoing process of deinstitutionalisation in most Western countries, we argue that more should be done to prevent unstable residential and care patterns in large groups of patients of the kind found in this study. Deinstitutionalisation should therefore be accompanied by flanking support programmes that can reduce the risk associated with frequent changes of care and residence. Our findings underline, for instance, the importance of integrating treatment and the prevention of substance use in treatment programmes.

Living independently is often seen as a desirable outcome in SMI patients but our results indicate that this aim should be reconsidered in some patients, who may be better off in sheltered housing. One could also argue that mental health care for those patients, who undergo highly frequent admissions and residential changes, needs improvement. This may imply that some patients need more support to live successfully in a stable situation. Future research should look at why the residential and care patterns of some patients are so turbulent and how we can help them to establish stable and safe homes.

## Abbreviations

BPRS-E: The Brief Psychiatric Rating Scale-Expanded
GLM: Generalised linear model
MAQ: Medication Adherence Questionnaire
MATE: Addictions for Triage and Evaluation
SMI: Severe mental illness
